# Finerenone in mildly reduced or preserved ejection fraction and chronic kidney disease: a narrative review

**DOI:** 10.1186/s43044-026-00722-x

**Published:** 2026-03-01

**Authors:** Muhammad Shaheer Bin Faheem, Hafiza Qurat Ul Ain, Aatka Rauf, Qasra Faheem, Own E Mohammad Najmi

**Affiliations:** 1 Karachi Institute of Medical Sciences, KIMS, Karachi, Pakistan; 2 CMH Multan Institute of Medical Sciences, Multan, Pakistan; 3 Fatima Memorial Hospital, Lahore, Pakistan

## Abstract

**Background:**

In patients with type 2 diabetes and chronic kidney disease (CKD) heart failure with preserved ejection fraction is associated with considerable morbidity and it has fewer treatment options available. HFpEF, which is characterized by increased cardiovascular risks and poor outcomes, continues to be a therapeutic challenge, particularly in older persons.

**Main body:**

A non-steroidal mineralocorticoid receptor antagonist (MRA), finerenone has excellent therapeutic potential in patients with HFpEF and CKD, notably those with diabetes type 2. Research indicate that Finerenone preferentially targets mineralocorticoid receptors associated with inflammation and fibrosis and that it also has an advantage over commonly used steroidal MRAs (e.g., spironolactone), which carry significant risks such as hyperkalemia, gynecomastia, and renal impairment. There are two phase 3 clinical trials namely FIDELIO-DKD and FIGARO-DKD demonstrating how effectively finerenone works in preventing the progression of renal damage and cardiovascular events in type 2 diabetic patients and in patients with CKD. In addition, the FINEARTS-HF study shows that hospitalizations for heart failure were reduced with use of finerenone and also the overall decline in individuals with HFpEF across all ages, particularly the older populations. Finally, Finerenone is found to be more appropriate for patients with complex medical histories because its safety profile shows fewer side effects compared to the traditional steroidal MRAs, as it has lower risks for electrolyte imbalances and renal function deterioration.

**Conclusion:**

This article addresses the safety, efficacy, mechanism of action, and various trials conducted on Finerenone and how it gained importance as an effective substitute in the management of HFpEF and CKD, decreasing morbidity and mortality risks while limiting adverse effects.

## Introduction

Heart failure is not an isolated clinical entity but rather a systemic condition characterized by key symptoms such as dyspnea, fatigue, and peripheral oedema, along with clinical signs including increased JVP (jugular venous pressure), and peripheral and pulmonary oedema. The condition arises from anatomical or cardiac working abnormalities, leading to elevated intra-ventricular impulses and diminished heart outflow at rest or during exertion. Chronic kidney disease (CKD) is a long-standing disorder involving gradual loss of kidney filtration function. Reduced kidney function leads to inadequate removal of fluid, electrolytes, and uremic toxins, triggering complications including hypertension, anemia, and increased risk of heart disease.

Heart failure with preserved ejection fraction (HFpEF), is a group of disorders marked by decreased ventricle compliance while preserving adequate systolic function, especially if the left ventricle’s EF is 50% or above. This condition is associated with decreased diastolic function and elevated blood levels of natriuretic peptides [1]. The pathophysiology of HFpEF is driven by systemic inflammation, neurohormonal and mineralocorticoid receptor activation (particularly in the setting of obesity and diabetes), hemodynamic dysfunction, and iron deficiency, which collectively interact with CKD to perpetuate a vicious cardiorenal cycle [2]. CKD and HFpEF frequently coexist, with chronic kidney disease present in approximately 40–60% of patients with HFpEF [3].

Finerenone is a non-steroidal, exclusive mineralocorticoid receptor antagonist (MRA) believed to have fewer side effects than steroidal mineralocorticoid antagonists. In this article, we will discuss safety, efficacy, mechanism of action and different trials done on Finerenone [4].

## Finerenone’s safety and effectiveness

Finerenone, which has anti-inflammatory and anti-fibrotic effects, has shown potential in preliminary studies for kidney illnesses and cardiovascular disorders. The risk of HFpEF surges significantly with age. In general, nearly half of all heart failure patients are diagnosed with HFpEF [5]. Even though the prevalence of heart failure related to age is declining, the decline is significantly less pronounced in heart failure with preserved ejection fraction in comparison to heart failure with lowered ejection fraction (HFrEF) [4]. Supplementary risk variables encompass high blood pressure, increased weight, and coronary artery disease (CAD). When age and other risk factors are excluded, both genders have a similar likelihood of developing heart failure with a maintained ejection fraction. However, females have a considerably lower risk of HFrEF. Co-morbidities are widespread in both types of heart failure, but they are more severe and fatal in HFpEF. Cardiovascular diseases account for the majority of deaths in individuals with HFpEF. Furthermore, in contrast to HFrEF (heart failure with reduced ejection fraction), HFpEF has a greater percentage of non-cardiovascular fatalities as well [5].

In the Phase 3 FIDELIO-DKD trial of adults with type 2 diabetes and chronic kidney disease receiving optimized ACEi/ARB therapy, finerenone significantly reduced progression of kidney disease compared with placebo. It also lowered the risk of major cardiovascular outcomes, including cardiovascular death, myocardial infarction, stroke, and heart-failure hospitalization [6]. 

Furthermore, In another phase 3 trial, FINEARTS-HF, of patients with heart failure with mildly reduced or preserved ejection fraction (LVEF ≥ 40%), finerenone consistently lowered the chance of cardiovascular mortality and total heart failure incidents across left ventricular ejection fraction (LVEF) categories: LVEF < 50%, ≥ 50 to < 60%, and ≥ 60%; p interaction = 0.70. No modification of the benefit of finerenone was observed throughout the spectrum of LVEF when evaluated as an ongoing parameter (p interaction = 0.28). Finerenone consistently minimized the overall amount of deteriorating heart failure incidents (continuous p interaction = 0.26). In those suffering from HFmrEF/HFpEF, finerenone decreased the risk for cardiac mortality and the incidence of more serious heart failure events, regardless of LVEF [7]. 

Heart failure with a slightly reduced cardiac ejection fraction (HFmrEF) of 40%-49% is a frequently overlooked kind of heart failure. The proportion of HFmrEF within the heart failure population ranges from 13% to 24%, indicating that approximately 0.8 to 1.6 million people living in the US are suffering from HFmrEF [8] Most significant heart failure (HF) studies traditionally omit patients with borderline ejection fraction. Despite the EF being a continuous metric with significant fluctuation, this comprehensive review posits that HFmrEF serves as a valuable classification for patients with heart failure, sharing key clinical characteristics with HFrEF, hence advocating for the reclassification of HFmrEF to heart failure with modestly lower EF. HFmrEF and HFpEF are becoming increasingly prevalent, and it is anticipated that these two types of heart failure will shortly surpass HFrEF to become the most common one worldwide. The intended target population for this course is primarily older individuals [9]. Consequently, a crucial contemporary medical challenge has emerged: developing effective treatments for HFmrEF, particularly in older age groups, to prevent heart failure episodes from getting worse and to optimize health. This challenge is made even more daunting by the possibility that heart failure and reduced ejection fraction in older adults make them frailer and feeble, and they are also likely to have numerous medical conditions that require them to take multiple medications. In addition to the fact that older patients have different pharmacodynamics and pharmacokinetics than younger persons, all of this raises concerns that the drug will not be tolerated well. Therefore, it is essential to assess the safety of introducing new medicines into the pre-existing regimens of older people. Without a thorough understanding of the efficacy and accessibility of innovative treatments, there is a significant risk of their underuse, as has been observed with several beneficial therapies in older persons [10]. 

### Safety and efficiency of steroidal mineralocorticoid receptor antagonists (sMRA) as compared to Finerenone

**Fig. 1:**
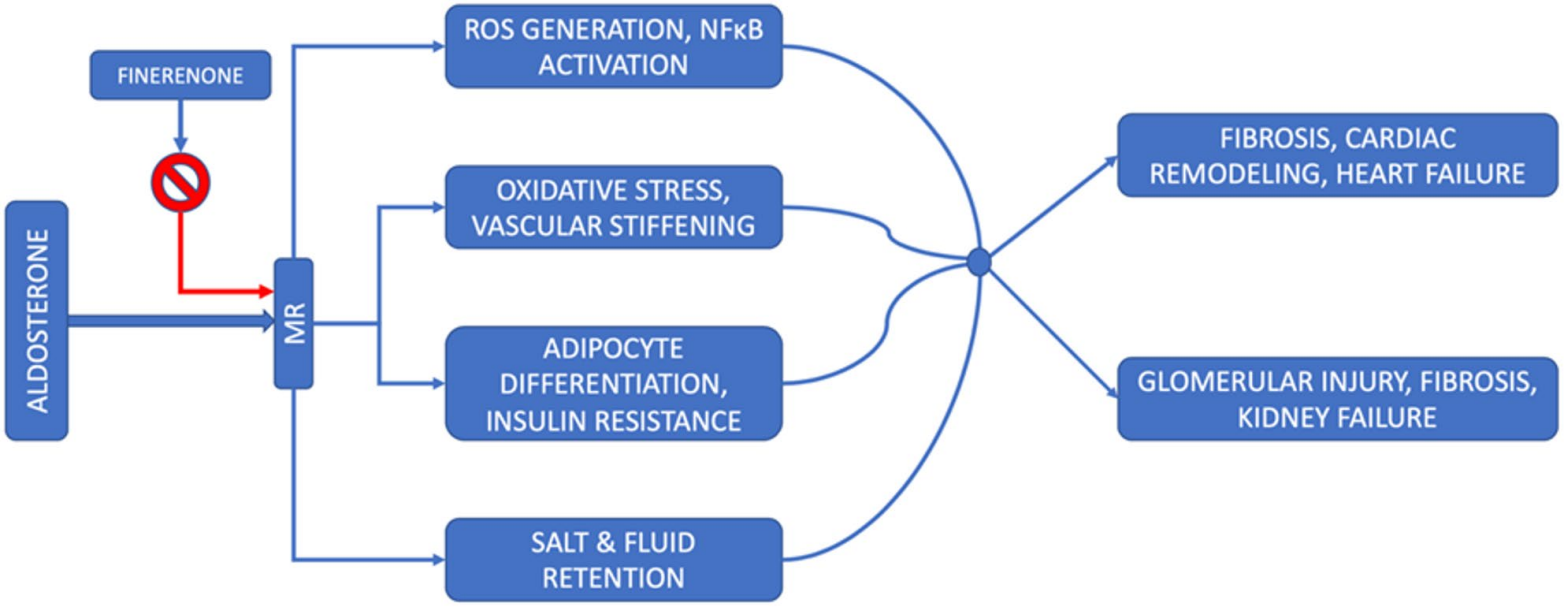
Mechanism of action of finerenone on the mineralocorticoid receptor (MR). [Abbreviations: MR, mineralocorticoid receptor; ROS, reactive oxygen species; NFκB, nuclear factor kappa B]

The use of steroidal mineralocorticoid receptor antagonists (MRA), namely eplerenone and spironolactone, in patients of chronic HFrEF is clinically advised. The balance between safety and efficacy in patients with a left ventricular ejection fraction of 40% or higher has not been fully comprehended. Moreover, the widespread use of steroid mineralocorticoid receptor antagonists has its limitations due to safety concerns such as hyperkalemia, gynecomastia, and renal dysfunction. Finerenone has more selectivity towards the mineralocorticoid receptor than spironolactone or eplerenone and can mitigate such risks due to its distinctive pharmacological properties. Its uniform dissemination in tissues of the kidneys and heart may decrease the likelihood of electrolyte imbalances. The following figure demonstrates how inhibiting the mineralocorticoid receptor (MR) lessens end-organ damage associated with chronic heart failure (HF) [4] (see Fig. 1). 

The therapeutic application of existing steroidal mineralocorticoid receptor antagonists (MRAs) is limited due to the risk of hyperkalemia, especially in individuals with impaired renal function. In contrast, Finerenone, demonstrates distinct efficacy and specificity in its action on the mineralocorticoid receptor.

## Clinical trials supporting finerenone in the management of heart failure

There remain several unresolved inquiries regarding the clinical application of finerenone, particularly concerning its safety and effectiveness, as well as its potential to impede the progression of CKD and also cardiovascular disease in individuals diagnosed with type 2 diabetes when administered in the highest tolerable dosage of renin-angiotensin system inhibition. These questions necessitate answers, prompting various clinical trials to examine effectiveness and safety.

Finerenone’s division in tissues and persistent cardio-renal end-organ safeguarding were compared to those of eplerenone, a steroidal MR antagonist, in two separate preclinical rat disease models. The cardiac and renal tissues of rats exhibited a uniform distribution of [C]-labelled Finerenone, which is Finerenone tagged with carbon-14 isotope, as revealed by quantitative whole-body autoradiography. Finerenone treatment protected rats subjected to deoxycorticosterone acetate and salt challenges from structural and functional renal and cardiac damages, even at doses that did not normalize systemic blood pressure. At equinatriuretic doses, compared to eplerenone, finerenone more effectively decreased the incidence of cardiac hypertrophy, lowered brain natriuretic peptide levels, and caused less proteinuria.

In rats with coronary artery ligation that caused heart failure, Finerenone 1 milligram per kg per day enhanced both systolic and diastolic function of the left ventricle while also reducing plasma levels of brain natriuretic peptide prohormones, whereas eplerenone (100 mg/kg/d) had no effect. According to the results of this preclinical study, finerenone may protect end organs while lowering the risk of electrolyte imbalance [4]. In two large-phase clinical trials, Finerenone helped over 13,000 patients with type 2 diabetes and individuals with chronic renal dysfunctions, showing its safety and effectiveness in mitigating the advancement of renal illness and averting cardiovascular incidents. In patients with heart failure with cardiorenal comorbidities, Finerenone showed promising results in reducing the negative consequences. In comparison to a placebo, when Finerenone was given to type 2 diabetic patients and patients with chronic renal disease, it caused a decreased likelihood of cardiac incidents and advancement of CKD [11]. When comparing finerenone therapy to placebo, there was slightly increased albuminuria in type 2 diabetic patients in stages 2 to 4 of Chronic Kidney Disease, or those with significantly increased albuminuria in stages 1 or 2 showed improved cardiovascular outcomes [12].

Over an average duration of three years of follow-up, it was seen that finerenone markedly lowered chances of hospitalization for heart failure by 22%, even though less than Ten per cent of patients in these trials had been diagnosed with heart failure, and patients with HFrEF with symptoms were omitted [13]. Finerenone was also found to decrease the incidence of medically significant renal and cardiovascular events in type 2 diabetes patients; among these patients, 40% had a higher estimated glomerular filtration rate of more than 60 ml per min per 1.73 m². The sole criteria used to identify these patients was albuminuria. Renal disease progression and risk of fatal cardiovascular outcomes were decreased with Finerenone in CKD and type 2 diabetes patients. In patients with type 2 diabetes, albuminuria evaluation helps identify the patients at risk, thus reducing cardiac and renal disease burdens [13].

The individuals diagnosed with type 2 diabetes, as well as with chronic renal dysfunctions at various stages, were included in two phase 3 trials to evaluate cardiovascular and renal outcomes, namely FIDELIO-DKD and FIGARO-DKD. to conduct a predetermined pooled effectiveness and safety analysis at the individual patient level across a wide range of chronic renal disease was the goal of fidelity analysis which offers robust assessments in terms of safety and effectiveness of Finerenone when compared to a placebo. The results of the FIGARO-DKD analysis emphasized that finerenone improves heart failure-related outcomes and reduces the occurrence of newly diagnosed heart failure in patients suffering from chronic renal dysfunctions and type 2 diabetes regardless of prior history of heart failure [14]. 

HFrEF and moderate CKD patients, when provided with 5 to 10 mg daily doses of Finerenone, demonstrated comparable efficacy to spironolactone at daily doses of 25 or 50 mg in lowering biomarkers associated with vascular stress. The biomarkers assessed included N-terminal pro-B-type Natriuretic peptide or NT-proBNP, renin and aldosterone levels, creatinine, high-sensitivity troponin and Blood Urea Nitrogen or BUN. These biomarkers assist healthcare practitioners in evaluating patients’ cardiac and renal conditions and monitoring treatment effectiveness [7].

Interestingly, finerenone was linked to a reduced occurrence of hyperkalemia and deteriorating kidney function. In a phase-2 trial, spironolactone at 25 or 50 mg once daily was found to be as effective as daily dose of 10 mg once or daily dosage of 5 mg twice of finerenone in lowering natriuretic peptides and albuminuria in patients with HFrEF and slight chronic renal disease. Raised levels of natriuretic peptides, used to measure heart failure, indicate increased cardiac stress, while elevated albumin excretion in urine indicates renal injury.

Furthermore, compared to spironolactone, Finerenone caused a lesser rise in blood potassium levels (hyperkalemia) without significant deterioration of renal function. In a greater phase two trial involving individuals exhibiting exacerbation of HFrEF with chronic renal disease and/or diabetes, the brief administration of Finerenone, which ranges from 2.5 mg to 20 mg once daily, proved as well tolerated in about the same number of patients as eplerenone (dose 50 mg once daily), in achieving a clinically significant decrease in levels of natriuretic peptides and similar effects on serum potassium levels [15].

Finerenone demonstrated a favourable safety profile and achieved a reduction of thirty percent or greater in serum levels of NT-pro BNP in a comparable proportion of patients as observed with eplerenone. A preliminary analysis showed a reduced risk for major cardiovascular and renal incidents even with an approved maximum dose of finerenone compared to eplerenone, thus achieving nominal statistical significance. This indicates that the results passed a preset threshold for significance (often *p* < 0.05) without fully accounting for multiple comparisons [16].

The latest FINEARTS-HF trial was a multicenter, hypothetical, randomly assigned, double-blinded, event-based study that analyzed the effectiveness and safety of Finerenone in comparison to a placebo in individuals diagnosed with HFmrEF/HFpEF. Several limitations must be considered when interpreting the FINEARTS-HF trial results. Even when there are limitations, this research presents one of the largest populations to date for evaluating patients diagnosed with HfmrEF/HFpEF across groups with diversity in age. In FINEARTS-HF, the known steroidal mineralocorticoid receptor antagonist (MRA) Finerenone proved to be equally efficient in reducing the primary results of total deteriorating heart failure incidents, including heart failure hospitalizations, immediate heart failure visits and cardiovascular mortalities, across all age groups.

A hospitalization for heart failure was defined as an admission with a primary diagnosis of heart failure that lasted for at least 24 h. An unplanned, urgent ER (emergency room)visit with a diagnosis of heart failure that necessitated intravenous diuretics, vasoactive medications, mechanical or surgical intervention, or both was defined as an urgent heart failure visit [17]. Finerenone enhanced overall health status compared to the placebo, as indicated by an elevation in the KCCQ-TSS score. At the same time, age had no impact on outcomes, finally, the report of safety and tolerability for Finerenone was consistent across all ages. The primary outcome benefit associated with finerenone was primarily influenced by a reduction in the worsening of heart failure events, while no notable advantage was observed in cardiovascular mortality. More importantly, the advantage of Finerenone seemed consistent across all age groups, with a lower risk in older individuals [7].

Another significant therapeutic goal in Heart failure (HF) is to reduce symptoms, boost physical capacity, and enhance overall health and well-being. In FINEARTS-HF, Finerenone produced a more considerable increase in KCCQ-TSS compared to placebo, with a constant elevation witnessed across all age groups, including very old patients. However, the condition of hypotension and hyperkalaemia occurred more often when dealt with Finerenone than with placebo. There was a notable linkage between age and treatment outcome on the mentioned parameters across all groups [7]. Kidney damage, hyperkalaemia, and the condition of hypotension are key concerns in older patients, leading to unwillingness to commence on medication or to discontinue therapy.

Certainly, multiple recent studies have determined that elderly patients use mineralocorticoid receptor antagonists (MRAs) at a considerably lower rate than younger people. For instance, in the Victoria trial, MRAs were administered to 81% of patients under 65, whereas only 56% of patients aged 75 years or older received them. Moreover, several clinical studies have shown that elderly patients with cardiovascular disease use fewer guideline-recommended therapies than their younger counterparts, raising concerns regarding potential ageism in routine prescribing practices. A comparison of some clinical trials that have been done on the safety and effectiveness of Finerenone is given in Tables 1 and 2 [18].

**Table 1:** Shows comparison of three phase 2 trials on determination of efficacy of finerenone

Phase 2 Trials			
Design Element	ARTS	ARTS-HF	ARTS-DN
Follow-up	4 weeks	3 months	3 months
Population	HFrEF and chronic kidney disease	HFrEF and T2D and/or chronic kidney disease	Chronic kidney disease and T2D
Primary endpoint	Serum potassium level changed	> 30% decline in NT-pro BNP	Change in UACR (urinary albumin to creatinine ratio)
Summary of primary results	Finerenone (10 mg one time daily and 5 mg twice daily) increased serum potassium more than spironolactone (Part B), albeit at a lower mean.	Comparable rates of NT-pro BNP decreases were reported in the Finerenone and Eplerenone groups.	Finerenone resulted in a dose-dependent reduction in UACR.

This table gives comparison between basic design elements of these trials and their primary results

These trials established that for the patients of Heart failure with chronic kidney disease or type two diabetes, finerenone proved to be a more safe and good addition to conventional treatment regimens

HFrEF Heart Failure with reduced ejection fraction, CKD Chronic Kidney Disease, T2D Type 2 Diabetes, NT -pro-BNP N-terminal pro Brain Natriuretic Peptide, CV Cardiovascular, UACR urinary albumin to creatinine ratio, MI myocardial infarction, GFR Glomerular filtration rate

**Table 2:** Shows comparison of three phase 3 trials on determination of efficacy of finerenone

Phase 3 Trials			
Design Element	FIDELIO-DKD	FIGARO-DKD	FINEARTS-HF
Sample size	5734	7437	6001
Follow-up	2.6 years (median)	3.4 years (median)	2.6 years (median)
Population	CKD and T2D	CKD and T2D	
Primary endpoint	Kidney failure, ≥ 40% decline in eGFR, or renal death	Cardiovascular mortality, non-fatal stroke, non-fatal MI or heart failure hospitalization	CV death and overall deterioration of HF incidents (hospitalizations/urgent visits)
Summary of primary results	Finerenone lowered the risk of CKD development by 18%.	Finerenone decreased the risk of cardiovascular events by 13%.	risk of cardiovascular death by 14% and worsening of heart failure by 20% reduced by finerenone

This table gives comparison between basic design elements of these trials and their primary results

These trials established that for the patients of Heart failure with chronic kidney disease or type two diabetes, finerenone proved to be a more safe and good addition to conventional treatment regimens

HFrEF Heart Failure with reduced ejection fraction, CKD Chronic Kidney Disease, T2D Type 2 Diabetes, NT -pro-BNP N-terminal pro Brain Natriuretic Peptide, CV Cardiovascular, UACR urinary albumin to creatinine ratio, MI myocardial infarction, GFR Glomerular filtration rate

FINE-REAL represents an inaugural observational study utilizing a non-steroidal MRA in a cohort of subjects having a chronic renal disorder and type 2 diabetes, anticipated to yield significant insights into managing chronic renal dysfunction linked to type 2 diabetes. FINE-REAL will guide decision-making regarding the initial administration of Finerenone for patients with type 2 diabetes and chronic kidney disease [19]. It exhibits an excellent safety record and demonstrates a reduced propensity for inducing hyperkalaemia relative to steroidal MRAs. It is presently utilized for patients having diabetic kidney disease and albuminuria. Numerous clinical studies are underway that may facilitate the broader application of finerenone to treat chronic renal dysfunction and heart failure [20].

Enhancing medication in chronic renal dysfunction that is associated with type 2 diabetes is essential to avoid or delay the transition to the last stage of renal dysfunction and decrease the frequency of cardiovascular outcomes. Numerous people suffering from chronic renal dysfunction get inadequate treatment due to the complexity of care needed, insufficient disease recognition by doctors and individuals receiving care, and underutilization of novel kidney-protective therapies. Finerenone is the US FDA and the European Medicines Agency’s approved non-steroidal selective mineralocorticoid receptor antagonist for the management of adult individuals with chronic renal dysfunction linked to type 2 diabetes. Clinical studies demonstrate that the risk of progression of cardiac and renal disease is markedly reduced by finerenone, as compared to a placebo, and it also shows a lower incidence of increased potassium levels than conventional steroidal mineralocorticoid receptor antagonists (MRAs). Before commencing treatment with finerenone, evaluating the baseline eGFR and blood potassium levels is essential. It is essential to weigh potential drug-drug interactions, observe potassium levels during follow-up, and coordinate pharmacotherapy changes among the patient’s care team [21, 22].

## Conclusion

Considering the various clinical trials performed to analyze the competence, safety, and effectiveness of Finerenone, it has been observed that Finerenone reduced the primary outcome and its components while improving symptoms across a broad age range. Moreover, Finerenone demonstrated consistent safety and tolerability, regardless of age.

The generalizability of the efficacy and safety results has long been questioned due to concerns that the patient populations chosen for clinical trials may not appropriately reflect the larger “real-world” population. Remarkably, numerous investigators have discovered that “real-world evidence” closely corresponds with the trial results, indicating that the results seen in clinical settings represent the larger patient population [7, 23].

Recent research has shown that aldosterone plays a broader role in nonepithelial activities, affecting inflammatory disorders, creation of collagen, necrosis and fibrosis. Emerging research suggests that the pathophysiological overactivation of mineralocorticoid receptors (MR) plays a pivotal role in chronic kidney disease progression and the risk of mortality and morbidity associated with it. Consequently, the antagonism of MR is currently under investigation as a viable treatment approach to mitigate the advancement of CKD [24–26]. 

## Data Availability

No datasets were generated or analysed during the current study.
